# Diel vertical migration of Arctic zooplankton during the polar night

**DOI:** 10.1098/rsbl.2008.0484

**Published:** 2008-10-23

**Authors:** Jørgen Berge, Finlo Cottier, Kim S. Last, Øystein Varpe, Eva Leu, Janne Søreide, Ketil Eiane, Stig Falk-Petersen, Kate Willis, Henrik Nygård, Daniel Vogedes, Colin Griffiths, Geir Johnsen, Dag Lorentzen, Andrew S. Brierley

**Affiliations:** 1 University Centre in SvalbardPb 156, 9171 Longyearbyen, Norway; 2 The Scottish Association for Marine Science, Dunstaffnage Marine LaboratoriesOban, Argyll PA37 1QA, UK; 3 Norwegian Polar Institute9296 Tromsø, Norway; 4 Bodø University College8049 Bodø, Norway; 5 Trondhjem biological station, Norwegian University of Science and Technology7491 Trondheim, Norway; 6 Gatty Marine Laboratory, University of St AndrewsSt Andrews, Fife KY16 8LB, UK

**Keywords:** diel vertical migration, circadian, polar night, Arctic, zooplankton, solar

## Abstract

High-latitude environments show extreme seasonal variation in physical and biological variables. The classic paradigm of Arctic marine ecosystems holds that most biological processes slow down or cease during the polar night. One key process that is generally assumed to cease during winter is diel vertical migration (DVM) of zooplankton. DVM constitutes the largest synchronized movement of biomass on the planet, and is of paramount importance for marine ecosystem function and carbon cycling. Here we present acoustic data that demonstrate a synchronized DVM behaviour of zooplankton that continues throughout the Arctic winter, in both open and ice-covered waters. We argue that even during the polar night, DVM is regulated by diel variations in solar and lunar illumination, which are at intensities far below the threshold of human perception. We also demonstrate that winter DVM is stronger in open waters compared with ice-covered waters. This suggests that the biologically mediated vertical flux of carbon will increase if there is a continued retreat of the Arctic winter sea ice cover.

## 1. Introduction

Many marine predators search for their prey visually, and their search efficiency is directly linked to ambient irradiance intensity (Yoshida *et al.* 2004). Consequently, herbivorous zooplankton have evolved a predator-avoidance behaviour (Hays 2003) known as diel vertical migration (DVM). Zooplankton ascend into food-rich surface waters during darkness and retreat to deeper waters during day (Fortier *et al.* 2001). These vertical migrations are integral to structuring pelagic communities and food webs because pelagic predators tune their behaviour to the migration pattern of their zooplankton prey (Hays 2003).

The absolute and relative changes in downwelling irradiance (*E*_d_, μmol photons m^−2^ s^−1^ at 400–700 nm) are considered the universal proximate trigger for DVM (Ringelberg 1999; Ringelberg & Van Gool 2003). There is an apparent absence of *E*_d_ during the polar night, defined here as the seasonal period when the Sun is more than 12° below the horizon, which humans perceive as being continuously dark. This, combined with the strong attenuation of light by sea ice, leads to the conventional paradigm that DVM should cease completely during winter in the Arctic marine environment. This understanding is entirely commensurate with the low food availability in winter (Smetacek & Nicol 2005) and the dormant overwintering strategies of some zooplankton species (Fortier *et al.* 2001; Falk-Petersen *et al*. 2008).

Most studies of DVM in the Arctic have focused on the period of midnight sun (Fortier *et al.* 2001; Blachowiak-Samolyk *et al.* 2006) or on the transition period between midnight sun and the overt day/night daily cycle (Blachowiak-Samolyk *et al.* 2006; Cottier *et al.* 2006; Falk-Petersen *et al*. 2008). There are few studies of biological processes during the polar night, largely owing to the logistical difficulties of sampling. One DVM study carried out in the Greenland Sea during winter failed to detect a clear vertical migration pattern during the period from November to January (Fischer & Visbeck 1993).

Here we use acoustic data from two coastal locations in Svalbard (Kongsfjorden and Rijpfjorden at 79° and 80° N, respectively) to test the hypothesis that there is no DVM behaviour during the polar night. Although both locations are at similar high latitudes, their oceanographic properties are representative of the extremes found throughout the Arctic. In combination, the results from these sites provide data that are relevant on a pan-Arctic scale (see the electronic supplementary material).

## 2. Material and methods

**Figure 1:**
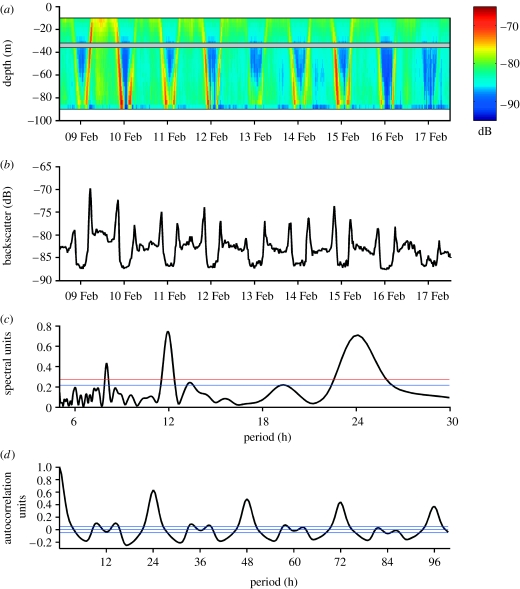
Derivation of the autocorrelation values for the periodicity of DVM using an example from the 30 to 34 m depth interval. (*a*) Backscatter values in the upper 100 m in Kongsfjorden. (*b*) Backscatter values from 30 to 34 m for the same period as in (*a*). Calculation of the dominant periods and strength of the periods in the backscatter data shown in (*b*) using (*c*) CLEAN spectral analysis and (*d*) autocorrelation routines in the MAZ software (Rosato & Kyriacou 2006). The spectrogram (*c*) shows three periodic components above the 99 per cent confidence limit (red line): 8 and 11.9 hours (peaks in the dusk/dawn ascent and descent patterns) and 24.1 hours (daily migration pattern). The correlogram (*d*) reconfirms the spectral analysis result with the strongest correlations at periods of 24 hours (the 48, 72 and 96 hours peaks are harmonics and ignored). The strength of the DVM signal is represented by the height of the 24-hour peak, which in this case is 0.64 and equates to highly synchronized activity.

Acoustic data were collected continuously at each study site using moored, upward-looking 300 kHz acoustic Doppler current profilers (ADCP). These data were analysed for temporal and spatial changes in the intensity of the acoustic backscatter signal to determine the presence, timing and amplitude of DVM. Backscatter intensity from a given depth in the water is positively correlated with the biomass of zooplankton there (figure 1). Temporal variation in echo intensity with depth is indicative of synchronized DVM (Cottier *et al.* 2006). The occurrence, synchronicity and depth range of DVM signals were determined using the circadian statistical analysis program CLEAN (Rosato & Kyriacou 2006). Since migrations occur as a movement of biomass through large parts of water column, the CLEAN spectral and autocorrelation analyses were applied to each depth layer independently (figure 1), thus enhancing the spatial and temporal resolutions with which the periodicity of these migrations were estimated (see the electronic supplementary material). A sediment trap on each mooring collected samples of zooplankton, which offer insight to the likely species composition of the migrating population (Willis *et al.* 2006).

## 3. Results

**Figure 2:**
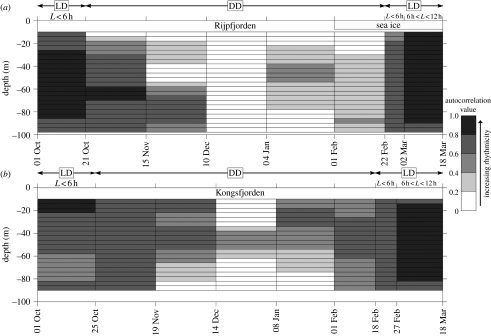
DVM signal strength and depth range during the winter in (*a*) Rijpfjorden and (*b*) Kongsfjorden. LD, light dark (difference between day and night); DD, dark dark (no apparent difference between day and night, Sun below the horizon). Each box represents a specific depth and time interval. The darker the shading at any particular depth, the more synchronized the 24-hour DVM signal. White boxes indicate no detectable DVM signal. All shaded boxes have a detectable DVM rhythm that is significant above a 99 per cent confidence interval.

The output from the CLEAN analysis (figure 2) summarizes the DVM signal. It shows 24-hour periodicity for each depth layer for defined periods throughout the polar night. From this, it is clear that zooplankton at both sites do perform DVM through most of the Arctic winter, even in those periods of the polar night with seemingly no diel changes in *E*_d_. Although the strongest DVM signal over the greatest vertical extent was detected at both sites in October and March when the Sun was above the horizon, giving a distinct contrast in irradiance between day and night, a near-continuous, albeit reduced, DVM signal was detected at both sites during the polar night (figure 2).

In Kongsfjorden, planktic organisms performed DVM from the middle of December until early January, within the period of the polar night, in a depth range limited to 30–60 m. However, from early January onwards the strength of the DVM pattern and its depth range increased in both fjords. At this time, a clear DVM signal was discernable throughout the upper 80 m in Kongsfjorden during a period with no apparent light trigger. From the beginning of February, when the Sun rose above the horizon, the DVM signal was strong throughout the water column at both sites. However, in Rijpfjorden where sea ice had formed, the DVM pattern showed a slightly reduced depth range and synchronicity compared with Kongsfjorden. By early March, with a strong day–night cycle of irradiance, active DVM was occurring in Rijpfjorden under a sea ice cover of up to 1 m thickness (J. Berge 2007, personal observation).

The overall acoustic backscatter signal was stronger in Kongsfjorden, indicating a larger total migrating biomass. Furthermore, sediment trap samples from Kongsfjorden contained more zooplankton individuals than those from Rijpfjorden (see the electronic supplementary material). The only group of species collected in traps throughout the winter were calanoid copepods. Note that during the early part of the winter, the depth range of DVM (shallower than 100 m) was above the depth of the traps moored at 100 m.

## 4. Discussion

Here we present the first evidence of DVM behaviour during the polar night, thereby highlighting a significant void in our current understanding of Arctic ecosystems. Precise identification of the migrants is not possible, but calanoid copepods, while rare, remain the only species collected from both fjords throughout the winter (see the electronic supplementary material). However, most calanoid species enter a state of diapause during the polar night (Conover 1988), and are thus expected to remain in the deeper parts of the water column. Future studies are thus needed to describe the winter community of high Arctic fjords as well as for exploring the adaptive value of winter DVM.

Throughout the study period, the DVM rhythm remained strictly within the circadian range. One possible explanation is that DVM is driven simply by an endogenous circadian clock. However, we reject this interpretation on the basis of the brief cessation followed by the re-emergence of a 24-hour DVM signal observed in Rijpfjorden in January (figure 2*a*). Such a spontaneous onset of DVM would require an exogenous trigger, and since most candidate triggers are either continuous (e.g. tides) or do not match the 24-hour DVM period (e.g. temperature) at either station (see the electronic supplementary material), we must look more closely at the apparently imperceptible variations in illumination.

During the periods with a day–night illumination cycle perceptive to the human eye (*E*_d_>0.17×0^−4^ μmol photons m^−2^ s^−1^), there was a significant (*p*<0.0001, *R*^2^=0.83) positive linear correlation between the hours of darkness and the duration of time that migrating organisms spent in the surface layer (see the electronic supplementary material). It is clear that *E*_d_ at this time is indeed the main proximate factor controlling the migration pattern. In the winter, when *E*_d_ is below the detection level for many standard irradiance meters, DVM was still detectable in both fjords. Irradiance data from the UNIS aurora station in Longyearbyen (www.unis.no) show a clear diel variation in solar background *E*_d_ in January, with an average maximum amplitude of 0.08×10^−4^ μmolphotons m^−2^ s^−1^, which is less than half the minimum detection level for the human eye. Hence, although not readily perceptible, diel cycles do in fact continue throughout the polar night and provide a potential proximate trigger for winter DVM. Furthermore, separate analysis of ADCP data 3 days prior to and after the time of full moon shows a shift in DVM periodicity from a 24-hour solar cycle towards a 25-hour lunar cycle. Such a shift, most clearly detectable in Kongsfjorden in December and January, strongly supports the idea that it is the relative change in *E*_d_, even during the polar night, which is the dominant trigger for the observed DVM.

These findings constitute a first step towards a new understanding of high Arctic marine ecosystems. Winter DVM might be of paramount importance in ecosystem dynamics and organism strategies for exploiting food sources. Our data suggest that Arctic zooplankton are able to detect and respond to subtle changes in irradiance, and imply that light is the key environmental trigger to maintain distinct DVM cycles during the polar night. The DVM signal was greater in Kongsfjorden (ice free) than Rijpfjorden (ice covered) perhaps owing to the additional attenuation of light by ice in Rijpfjorden. As DVM is a critical component of the ‘biological pump’ that draws organic carbon and atmospheric CO_2_ into the ocean interior (Longhurst 1989; Tarling & Johnson 2006), our results imply that an increased activity of the biological pump in Arctic waters could be expected in response to the predicted reduction in winter sea ice cover (Comiso 2006).

## Supplementary Material

EMS Berge *et al*. Winter DVM revised.doc Main body of text including fig captions and references

Map of study area EMS figure 1

Temperature profiles and light climate at the two locations EMS figure 2

Figure 3 in EMS updated.doc EMS figure 3

Relationship between hours of darkness and time spent in the surface EMS figure 4
