# Supplementary material for: Diel vertical migration of Arctic zooplankton during the polar night
Source: Biol Lett. 2008 Oct 23;5(1):69–72. doi: 10.1098/rsbl.2008.0484 (PMC2657746; doi:10.1098/rsbl.2008.0484)
Supplement: EMS Berge et al. Winter DVM revised.doc — Main body of text including fig captions and references [file rsbl20080484s09.doc]

**ELECTRONIC SUPPLEMANTARY MATERIAL**

**The study sites**

This study was conducted at two coastal sites around the archipelago of Svalbard (Figure ESM 1) in two fjords, Kongsfjorden and Rijpfjorden. Kongsfjorden is west facing and has a strong pulsed influx of relatively warm Atlantic Water (Cottier et al. 2005; Cottier et al. 2007). Its resident zooplankton population contains both Arctic and boreal species (Hop et al. 2002). Kongsfjorden has remained ice free for the last three winters (2005 to 2008). During the winter 2006-07 the water temperature never fell below 0°C (Figure EMS2). In contrast, Rijpfjorden is a north-facing fjord that opens towards the Arctic Ocean. There is little evidence of any influence of advected warm Atlantic water there, and its zooplankton community is dominated by true Arctic species (Falk-Petersen et al. 2008). Rijpfjorden experiences annual fast ice cover: in 2007 the fjord was ice covered from February 2007 until the middle of July (Figure EMS2). During the study period the water temperature in Rijpfjorden remained at -1.8°C throughout the water column..

**Methods**

*Instrumentation*

Two moorings with temperature and salinity loggers, sediment traps, fluorometers and 300 kHz acoustic Doppler current profilers (ADCP) were deployed in approximately 200 m water depth in Kongsfjorden and Rijpfjorden in June 2006 and September 2006, respectively. Both moorings were recovered in August 2007. The ADCPs and sediment traps were mounted at approximately 100 m depth; temperature and salinity loggers were spaced evenly? through the water column. The ADCPs transmit an acoustic pulse through the water column and and record the echo. The intensity of the backscattered signal returning from each discrete depth interval (bins) is related to the zooplankton biomass present within each bin. The ADCPs recorded velocity and backscatter intensity data every 20 minutes, averaged over 20 pings, in 22 depth layers (bin-depth 4 m). A data quality check was performed based on signal to noise ratio using the instruments’ proprietory correlation index. Vertical velocity values were corrected and the raw echo intensity data were converted to absolute volume backscatter (Sv, dB) (Cottier et al. 2006; Tarling et al. 2002). The size of zooplankton detected by the ADCPs is likely to span from the smallest *Calanus* spp and upwards.

DVM behaviour as described in our paper is based on data from the sediment trap and upward-looking ADCPs. Acoustic backscatter data wereanalysed to determine patterns of temporal and spatial changes in the echo intensity.. The classic acoustic signature of DVM is comprised of a layer of high backscatter ascending to the surface at dusk followed by a descent into deeper water around dawn . The backscattering intensity is indicative of the fraction of the zooplankton population migrating through the water column, and gives a measure of the degree of synchronicity exhibited by the population (Cottier et al. 2006). The occurrence, synchronicity and depth range of a DVM signal was determined here using the circadian statistical analysis algorithm CLEAN (Rosato & Kyriacou 2006). Sediment trap samples were analysed following previously-described protocols (Willis et al. 2006). All zooplankton were picked out of the samples, and all specimens identified to the species level. A summary of the most abundant species is presented in Figure EMS3.

*Statistical data analyses*

The CLEAN algorithm was implemented using the MAZ software package (Rosato & Kyriacou 2006) and was used to analyse the cyclicity of the ADCP backscatter data. The CLEAN algorithm used autocorrelation functions to calculate the most significant DVM period and its “strength”. Time epochs (Figure EMS4) to be analysed were chosen on the basis of the duration of daylight, twilight and ice cover. For each time epoch and ADCP depth bin CLEAN determined the most significant period (the highest spectrogram peak exceeding the 99% confidence interval). For diel (circadian) processes this will be near 24 hours. For each significant period identified, autocorrelation analysis was then used to resolve the “strength” of that period. Perfectly correlated DVM activity would produce a near 24 hour period “strength” value of 1. Values less than this reflect decreasing period “strength” as a result of increasing signal to noise ratio (either as the DVM is generally reduced throughout the time period in question or as the DVM differs in strength within the same period). Since the autocorrelation output represents normally distributed correlation coefficients, which are dimensionless (Levine et al. 2002), a direct comparison can be made between all time epochs and depth bins.

A linear regression between hours of darkness and time spent in the surface layers of the water column (Figure EMS4) was carried out based on *in situ* measurements of ambient light (Figure EMS2) and backscatter data from the ADCPs.

Cottier, F. R., Tverberg, V., Inall, M. E., Svendsen, H., Nilsen, F. & Griffiths, C. 2005 Water mass modification in an Arctic fjord through cross-shelf exchange: The seasonal hydrography of Kongsfjorden, Svalbard. *J. Geophys. Res.-Oceans*, 110, doi:10.1029/2004JC002757.

Cottier, F. R., Tarling, G. A., Wold, A. & Falk-Petersen, S. 2006 Unsynchronised and synchronised vertical migration of zooplankton in a high Arctic fjord. *Limnol. Oceanogr.*, In Press.

Cottier, F. R., Nilsen, F., Inall, M. E., Gerland, S., Tverberg, V. & Svendsen, H. 2007 Wintertime warming of an Arctic shelf in response to large-scale atmospheric circulation. *Geophys. Res. Lett.*, 34, doi:10.1029/2007GL029948.

Falk-Petersen, S., Leu, E., Berge, J., Kwasniewski, S., Nygård, H., Røstad, A., Keskinen, E., Thormar, J., von Quillfeldt, C., Wold, A. & Gulliksen, B. 2008 Vertical migration in high Arctic waters during Autumn 2004. *Deep-Sea Research II*, doi:10.1016/j.dsr2.2008.05.010.

Hop, H., Pearson, T., Hegseth, E. N., Kovacs, K. M., Wiencke, C., Kwasniewski, S., Eiane, K., Mehlum, F., Gulliksen, B., Wlodarska-Kowalczuk, M., Lydersen, C., Weslawski, J. M., Cochrane, S., Gabrielsen, G. W., Leakey, R. J. G., Lønne, O. J., Zajaczkowski, M., Falk-Petersen, S., Kendall, M., Wängberg, S.-A., Bischof, K., Voronkov, A. Y., Kovaltchouk, N. A., Wiktor, J., Poltermann, M., di Prisco, G., Papucci, C. & Gerland, S. 2002 The marine ecosystem of Kongsfjorden, Svalbard. *Polar Research*, 21, 167-208.

Levine, J. D., Funes, P., Dowse, H. B. & Hall, J. C. 2002 Signal analysis of behavioural and molecular data. *BMC Neuroscience*, 3.

Rosato, E. & Kyriacou, C. P. 2006 Analysis of locomotor activity in Drosophila. *Nature Protocols*, 1, 559-568.

Tarling, G. A., Jarvis, T., Emsley, S. M. & Matthews, J. B. L. 2002 Midnight sinking behaviour in *Calanus finmarchicus*: a response to satiation or krill predation? *Mar. Ecol.-Prog. Ser.*, 240, 183-194.

Willis, K. J., Cottier, F. R., Kwasniewski, S., Wold, A. & Falk-Petersen, S. 2006 The influence of advection on zooplankton community composition in an Arctic fjord (Kongsfjorden, Svalbard). *J. Mar. Syst.*, 61, 39-54.

**EMS figure legends**

Figure EMS1. Map of Svalbard and the Fram Strait with the two study sites indicated by star symbols. Bathymetry lines are given for 250, 500, 1000 and 2000m depth.

Figure EMS2. 12 month Temperature profiles for Rijpfjorden (a) and Kongsfjorden (b). The black contour lines (3^o^C) indicate the presence of warm Atlantic Water, although the salinity of the water masses in Rijpfjorden did not carry a strong signature of Atlantic Water. (c) *in situ* light measurements in air at the surface at local noon from both study sites (not intercalibrated data).

Figure EMS3. Zooplankton species collected in the sediment traps in Ripfjorden and Kongsfjorden. Contents are plotted as individuals per trap per week. Sampling periods per trap differed between the fjords, ranging from two months to one week (indicated by the grey lines).

Figure EMS4. Linear regression of hours of darkness (each day) and the number of minutes the zooplankton remain in surface layers (upper 10m).
