# Supplementary figures and images for: Diel vertical migration of Arctic zooplankton during the polar night
Source: Biol Lett. 2008 Oct 23;5(1):69–72. doi: 10.1098/rsbl.2008.0484 (PMC2657746; doi:10.1098/rsbl.2008.0484)

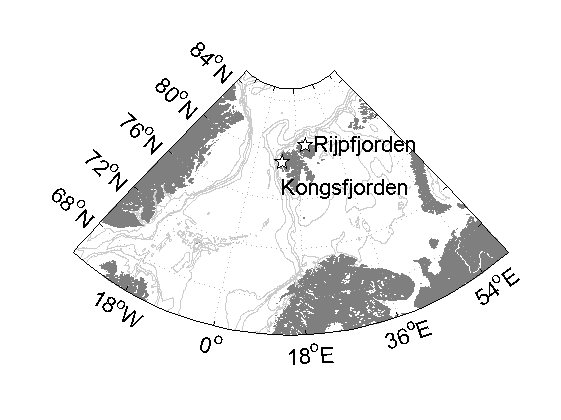


EMS Figure 1

Supplement: Map of study area — EMS figure 1 [file rsbl20080484s10.doc]

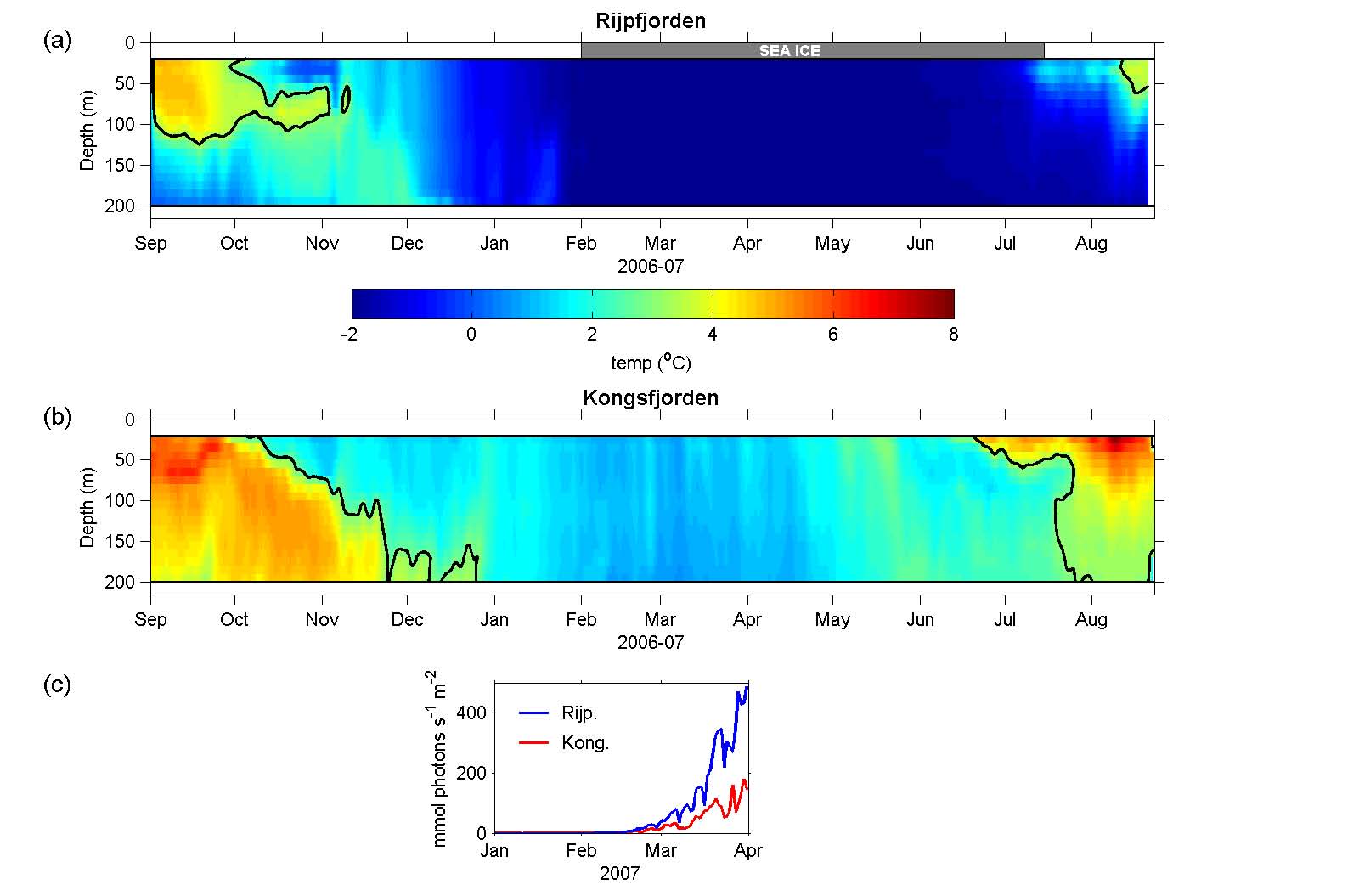


EMS Figure 2

Supplement: Temperature profiles and light climate at the two locations — EMS figure 2 [file rsbl20080484s11.doc]
