# Supplementary material for: Diel vertical migration of Arctic zooplankton during the polar night
Source: Biol Lett. 2008 Oct 23;5(1):69–72. doi: 10.1098/rsbl.2008.0484 (PMC2657746; doi:10.1098/rsbl.2008.0484)
Supplement: Relationship between hours of darkness and time spent in the surface — EMS figure 4 [file rsbl20080484s13.doc]

EMS Figure 4
