# Supplementary material for: Diel vertical migration of Arctic zooplankton during the polar night
Source: Biol Lett. 2008 Oct 23;5(1):69–72. doi: 10.1098/rsbl.2008.0484 (PMC2657746; doi:10.1098/rsbl.2008.0484)
Supplement: Supplementary file 4 [file rsbl20080484s12.doc]

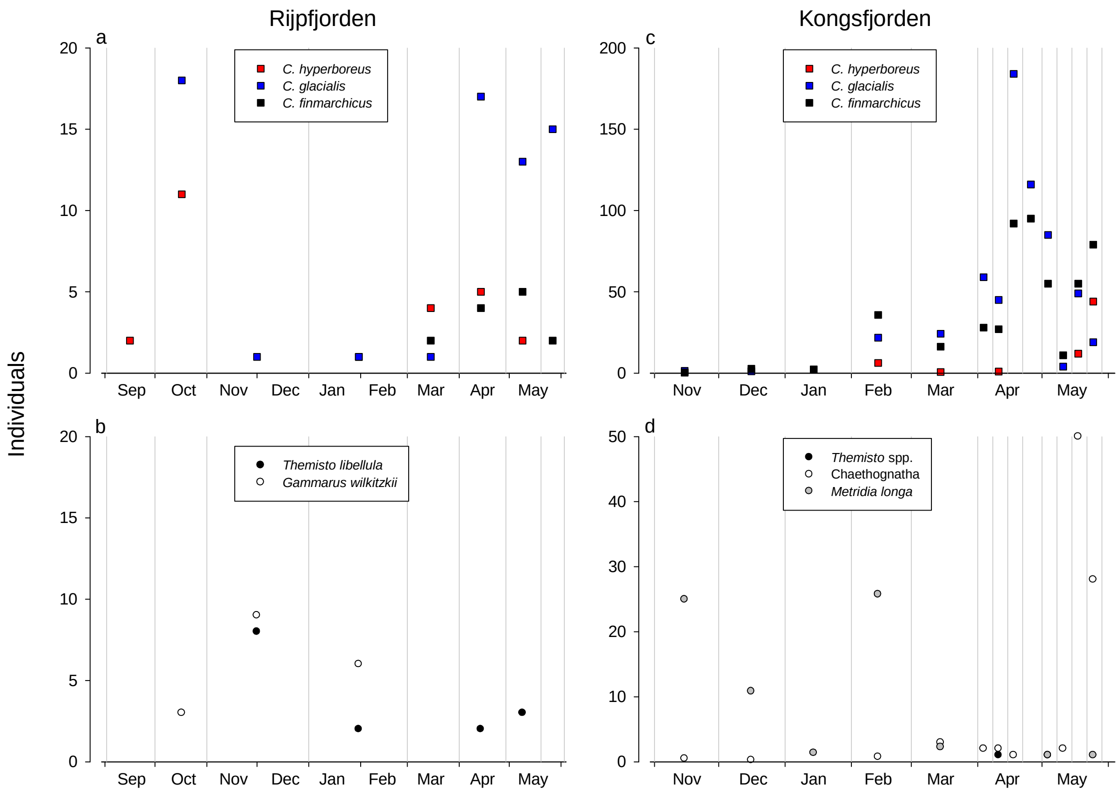

Rijpfjorden
Kongsfjorden
a
b
c
d
Individuals
C. hyperboreus
C. glacialis
C. finmarchicus
Themisto libellula
Gammarus wilkitzkii
Themisto spp.
Chaethognatha
Metridia longa
Sep
Oct
Nov
Dec
Jan
Feb
Mar
Apr
May
0
5
10
15
20
50
100
150
200
30
40
